# Shielding the Mind With Flow: Attention Allocation and Auditory Event‐Related Potentials Under Varying Mental Workload

**DOI:** 10.1111/ejn.70283

**Published:** 2025-10-20

**Authors:** Katharina Lingelbach, Anna Vorreuther, Elias Moll, Mathias Vukelić

**Affiliations:** ^1^ Applied Neurocognitive Systems Fraunhofer Institute for Industrial Engineering IAO Stuttgart Germany; ^2^ Applied Neurocognitive Psychology Carl von Ossietzky University Oldenburg Germany; ^3^ Applied Neurocognitive Systems, Institute of Human Factors and Technology Management IAT University of Stuttgart Stuttgart Germany

**Keywords:** electroencephalography (EEG), flow, game‐based paradigm, implicit oddball, mental workload, multivariate pattern analysis (MVPA)

## Abstract

Attention allows individuals to prioritize and effectively process relevant information while ignoring task‐irrelevant distractions. It plays a critical role in task performance, learning, and creativity. This study examines how varying levels of workload influence auditory attention, cognitive resource allocation, and the experience of flow. Thirteen participants engaged in a game‐based electroencephalographic study designed to induce states of mental underload, overload, and flow. To assess available attentional resources, an implicit auditory oddball task was integrated as a secondary task. Spatiotemporal cluster analyses revealed significant differences in event‐related potentials when comparing flow and overload to underload. Multivariate pattern analysis successfully decoded all three conditions above chance level, particularly in centroparietal regions. Subjective measures, including the NASA Task Load Index and Flow Short Scale, along with behavioral performance metrics, confirmed the effective induction of flow and distinct levels of workload. Notably, participants demonstrated significantly higher performance and subjectively perceived valence during the flow condition compared to the overload condition, albeit with similar levels of neural engagement. Our results support the notion that experiencing flow may act as a “shielding mechanism,” enhancing the effective allocation of attentional resources to the game and improving task engagement and performance efficiency.

## Graphical abstract

We used electroencephalography to investigate how different mental workloads affect auditory attention, cognitive resource allocation, and the experience of flow. Event‐related potentials and multivariate pattern analyses revealed distinct neural patterns for states of underload, overload, and flow in a game‐based task. Flow enhanced task performance and engagement, acting as a “shielding mechanism” for attentional resources.

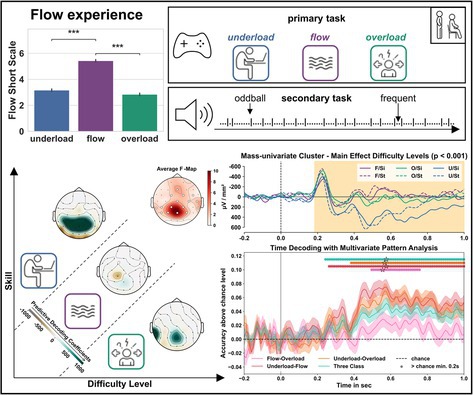

## 1 Introduction

The pleasurable experience of flow is facilitated by a balance between individual skill and the challenge of the task, which effectively utilizes cognitive resources (Nakamura and Csikszentmihalyi 2014). Flow is described as a state of deep immersion wherein the individual becomes entirely absorbed in the task at hand (Jackson et al. 2008; Sadlo 2016), while maintaining high levels of concentration and focus (Csikszentmihalyi 2014). This deep immersion is characterized by intensely focused attention on the task at hand, which likely supports a shielding mechanism against task‐irrelevant information (i.e., distractors; Dietrich 2004; Gold and Ciorciari 2020; de Sampaio Barros et al. 2024; Weber et al. 2009). Importantly, the state of flow is proposed to be linked to the dopaminergic neural system (Kotler et al. 2022; de Manzano et al. 2013; van der Linden et al. 2021). This link to the neural reward system likely distinguishes flow‐based states of focused attention from those induced by high task difficulty and workload, contributing to feelings of positive valence and reduced experienced workload (for review, see Alameda et al. 2022).

Both psychological (Ellis et al. 1994; Engeser and Rheinberg 2008; Keller and Bless 2008; Schaffer and Fang 2016; Tse et al. 2022) and neuroscientific studies (Goldberg et al. 2006; Huskey et al. 2018; Ju and Wallraven 2019; Klasen et al. 2012; Leroy and Cheron 2020; Lu et al. 2025; Peifer et al. 2014; Raichle et al. 2001; Sadlo 2016; de Sampaio Barros et al. 2024; Ulrich et al. 2016; Ulrich et al. 2014; Yoshida et al. 2014) agree that flow strongly depends on the balance between task difficulty and individual skill level. Consequently, designing experimental tasks tailored to each participant's skill level and preferences (Asakawa 2010; de Manzano et al. 2013; Ullén et al. 2012), while preserving replicability and comparability, is critical in flow research (see Durcan et al. 2024 for recent review). Game‐based paradigms offer a powerful tool by providing an engaging and intuitive framework that more closely mirrors real‐world decision‐making and behavior than traditional laboratory experiments (Allen et al. 2024; see Engeser and Rheinberg 2008; Keller and Bless 2008; Khoshnoud et al. 2020 for detailed reviews). By offering immersive experiences with easily scalable difficulty tailored to individual skills, game‐based paradigms foster intrinsic motivation to engage with the task, thereby facilitating the induction and maintenance of flow (de Sampaio Barros et al. 2024, cf. Durcan et al. 2024; Gold and Ciorciari 2020).

Subjective assessments via questionnaires enable the confirmation of flow experiences during a gaming period (Ellis et al. 1994; Schaffer and Fang 2016; Tse et al. 2022). However, as these measures are typically administered post hoc, they are less suited for capturing ad hoc immersion and focused attention during flow. A common approach is to record behavioral and neurophysiological measures such as electroencephalography (EEG) during flow experiences. To investigate the neurophysiological correlates of immersion and focused attention during flow, a secondary task can be introduced via a different sensory modality to probe attentional shifts from the primary task (Bombeke et al. 2018; Huskey et al. 2018; Maclin et al. 2011; Núñez Castellar et al. 2019). One such dual‐task paradigm is the auditory oddball paradigm, in which a frequent sound is played during the ongoing primary task. This frequent sound is randomly interspersed with one or more different rare “oddball” sounds at irregular intervals (Squires et al. 1975). Auditory event‐related potentials (ERPs), such as the P300 wave, have been associated with the attentional resources allocated to the oddball sound (Luck 2014; Polich 2007). The participants' task is typically to react to these oddball sounds immediately in some way while maintaining focus on the primary task (Bombeke et al. 2018; Huskey et al. 2018; Núñez Castellar et al. 2019). Maclin et al. (2011) investigated the effects of training on focused attention using a novel oddball dual‐task paradigm, wherein participants provided only an indirect response in the secondary task (i.e., silently counting the oddball sounds). Due to an improved preservation of task immersion within the dual‐task setup, the paradigm likely facilitates the investigation of focused attention and shielding mechanisms during a flow state.

To date, no study has employed this paradigm in flow research to examine how attentional allocation is influenced by the flow experience compared to varying levels of mental workload in a dual‐task setup involving an indirect response in the secondary task. Identifying and replicating neurophysiological signatures of flow in these scenarios could yield important indicators of the flow state and help detect environmental changes that may jeopardize its maintenance.

We recorded EEG during a primary game‐based task to induce different workload states (underload, flow, and overload), while simultaneously presenting an auditory oddball task. Participants indirectly attended the oddball stimuli by silently counting them, without any overt motor response. We investigated differences in attentional allocation in mass‐univariate spatiotemporal permutation‐based clustering analyses of evoked potentials, as well as in a multivariate subject‐wise approach to decode the different mental workload states time point–by–time point using supervised machine learning.

We expected subjective and behavioral measures to reflect differences between the flow condition and the overload and underload conditions. Specifically, it was hypothesized that reports of subjective flow experience and primary task performance would be significantly higher in the flow condition compared to the other conditions. On a neurophysiological level, we assumed that both flow and overload, compared to underload, would decrease the amplitude of the ERP component P300. The experience of flow was expected to lead to deep immersion and focused attention that shields from the auditory tone, while overload was assumed to deplete cognitive resources required to perform both tasks. We were particularly interested in differentiating flow from overload, hypothesizing that flow would be associated with the greatest reduction in ERP amplitudes and attenuated attentional shifts. This hypothesis links the neurophysiological markers of flow to its subjective and behavioral signatures, characterized by reduced perceived workload, positive valence, and enhanced performance.

Finally, we were interested in whether ergonomic positions commonly implemented in office workplaces (i.e., standing and sitting) would influence flow experience. Previous studies found mixed results of ergonomic position on factors influencing flow experience, including reports of no effect on cognitive performance (Russell et al. 2016), a positive effect on task engagement (Finch et al. 2017), and workload (Ghesmaty Sangachin et al. 2016), and mixed effects on productivity outcomes (Karakolis and Callaghan 2014). Therefore, we tested both positions in a counterbalanced within‐subjects design.

## 2 Methods

### 2.1 Participants

Fifteen participants were recruited for the study. Two were excluded due to technical errors during the recording sessions, leaving data from 13 participants for the analyses (*M*_age_ = 27.73 ± 3.82; range: 20 to 35 years; 7 female and 6 male participants). Prior to being invited to the laboratory, participants were screened for exclusion criteria using an online survey. All participants had to have normal or corrected‐to‐normal vision and adequate language skills, be right‐handed, and report no history of mental, neurological, or cardiovascular disease or use of centrally acting substances. They signed a written informed consent before starting the experiments and received financial compensation after completion. The study was conducted in accordance with the tenets of the Declaration of Helsinki and approved by the local ethics committee of the Medical Faculty of the University of Tübingen, Germany (ID: 827/2020BO1).

### 2.2 Experimental Procedure

**FIGURE 1:**
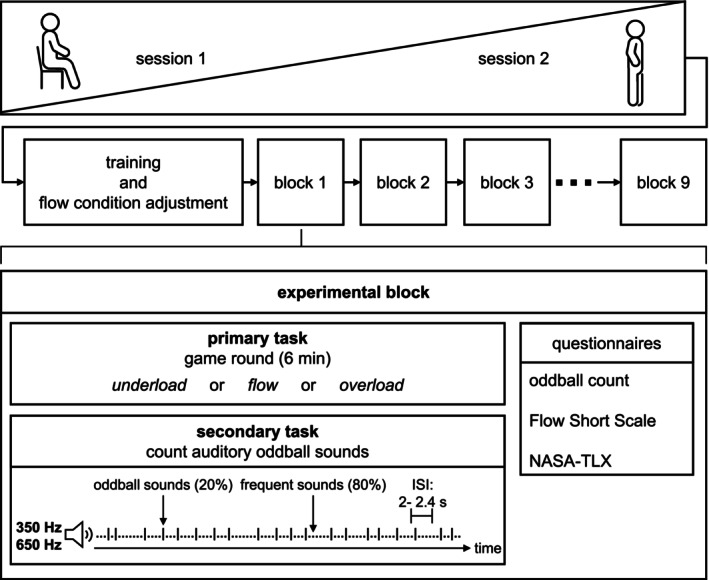
Experimental procedure. *Note:* Each participant completed two experimental sessions in different ergonomic positions (sitting or standing). Each experimental session consisted of a pre‐experimental training and familiarization phase followed by nine experimental blocks. Each block entailed a dual‐task setup during which participants performed a primary game‐based task that was presented in three difficulty levels (underload, overload, and flow) and a secondary silent auditory oddball counting task. Each block was completed by reporting the oddball count and completing the Flow Short Scale and NASA‐TLX. Abbreviations: ISI, interstimulus interval; NASA‐TLX, NASA Task Load Index.

After giving written informed consent, participants were equipped with a 32‐channel gel‐based EEG cap and electrooculography (EOG) electrodes (for details, see Section 2.4.2). Each participant completed two experimental sessions 1 week apart at the same time of day, one in a standing position and one in a sitting position (see Figure 1). The order of ergonomic positions was counterbalanced across participants. The experiment commenced with a training and familiarization phase (for details, see Section 2.3). This was followed by nine experimental blocks of approximately 6 min each, randomized across participants. During a block, participants were engaged in a primary task consisting of a computer game while simultaneously performing an auditory oddball counting task. Two sinusoidal sounds (350 and 650 Hz) were presented at jittered interstimulus intervals (2–2.4 s) via speakers at an output volume of 45–50 dB. Prior to the experimental blocks, participants were familiarized with both sounds of the secondary task and informed which sound was their target sound for silent counting (i.e., the oddball). The choice of target sound was counterbalanced across participants, and the target sound was presented 20% of the time. Participants were instructed to count its occurrences over the course of an experimental block. Each block varied in the difficulty of the primary task to induce one of the three mental workload conditions (underload, overload, and flow; for details, see Section 2.3).

### 2.3 Game Design

**FIGURE 2:**
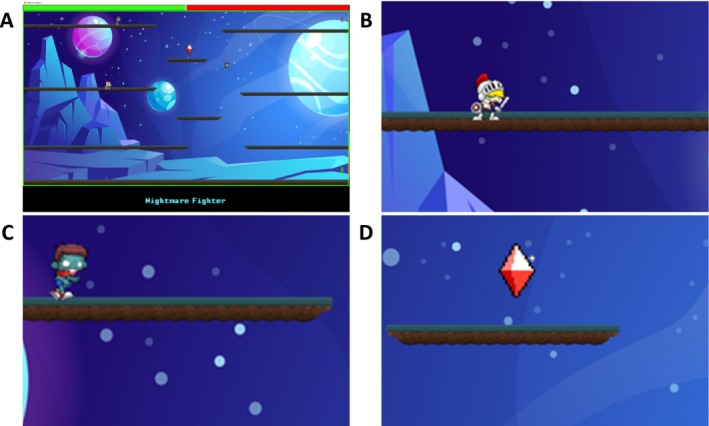
Screenshots of the game task. *Note:* (A) The game's layout with the performance indicator filling up in green at the top of the screen. (B) The hero character. (C) An enemy character. (D) A red diamond that could be collected for points.

The game was designed as a two‐dimensional arcade‐style platformer utilizing the *pygame* library (Pygame Community) and assets by O'Reilly (Eramo 2021; see Figure 2). The game ran at 60 frames per second with background music at 30–39 dB. Participants used keyboard input to control a character and collect diamonds while avoiding enemies. The landscape allowed screen wrapping, meaning that objects that left the screen at one edge reappeared at the opposite edge. Gravity made objects fall to lower levels, although the bottom corners were connected to the top corners to prevent accumulation at the bottom.

Participants were instructed to score as many points as possible and received feedback on their performance via a bar that filled up at the top of the screen. In addition, the player's character could lose points for penalized actions (e.g., contact with an enemy character or losing a diamond to an enemy; see Figure 2). Performance was calculated as a relative score based on points gained and lost.

The number of spawning enemy characters was used to induce different difficulty levels. To induce an underload difficulty level, the spawn rate of enemies was set to 45 s per enemy with a maximum of three entities at once. For overload, the spawn rate was set to 1 s with a maximum of 180 entities. These levels were established in a behavioral pilot study (*N* = 10, *M*_age_ = 26.5 ± 2.58; range: 20 to 29 years; 7 female and 3 male participants; 9 right‐handed; for details, see Table A10). To induce a flow experience, the difficulty level was adapted during gameplay to each participant individually. Prior to the experimental blocks, participants played the game and indicated which enemy spawn rate they found enjoyable. They could adjust the spawn rate themselves via button presses indicating “less time” or “more time” until the next enemy appeared. During the experimental blocks, the flow condition level started with a spawning rate of 2 s less than indicated by the participant. To consider the variable performance abilities of participants, two additional rules were implemented in the flow condition: (1) The maximum number of enemy entities could not exceed five to ensure that the flow condition would not exponentially increase in terms of challenge; (2) regardless of the current spawn rate, if the screen was empty for 6 s, an enemy would spawn to avoid underload in case of a rapid increase in skill of the participant relative to the challenge.

### 2.4 Data Acquisition

#### 2.4.1 Behavioral and Subjective Data

After each experimental block, participants were asked to indicate the counted oddballs during the block. False positives and misses were not distinguished; hence, the question was not used to evaluate the sensitivity/specificity of behavioral performance and instead primarily functioned to reiterate the importance for participants to stay engaged in the secondary task as well. Additionally, they filled in two questionnaires: the NASA Task Load Index (NASA‐TLX; Hart and Staveland 1988), which assesses perceived mental load on a scale ranging from 0 to 100, and the Flow Short Scale (FSS; Rheinberg et al. 2003), which measures overall skill level and the balance between challenge and skill level to estimate the subjective flow experience. The FSS consists of 13 items, of which 10 are answered on a 7‐point Likert scale (1: “not at all,” 7: “very much”) and three are indexed on a 9‐point scale (1: “easy/low/too low,” 9: “difficult/high/too high”).

#### 2.4.2 Neurophysiological Data

We recorded scalp EEG potentials using the international 10–20 system with 32 electrodes (actiCAP, Brain Products GmbH, Germany). The FCz was used as a common reference, and the EEG was grounded to the Fpz (for detailed layout, see Figure A1). The electrode impedance was kept below 20 kΩ at the beginning of each session. In addition to the EEG signals, EOG and electrocardiographic (ECG) signals were recorded (BrainAmp, Brain Products GmbH, Germany). To measure eye movements, four Ag/AgCl EOG electrodes were placed above and below the left eye (vertical, blinking) and on the outer canthi of both eyes (horizontal, saccades). To measure cardiac activity using the Einthoven technique, three Ag/AgCl electrodes were placed on the left clavicle, sternum, and left elbow (ground). The data were digitized at 250 Hz, high‐pass filtered with a time constant of 10 s, and stored for off‐line analysis using BrainVision Recorder software (Brain Products GmbH, Germany).

### 2.5 Analyses

All analyses were performed in Python.

#### 2.5.1 Behavioral and Subjective Data

A repeated‐measures ANOVA (rmANOVA) was used to evaluate the main and interaction effects of the within‐subjects factors *difficulty level* (underload, flow, overload) and *ergonomic position* (standing, sitting). The effect was evaluated by subjective scales (NASA‐TLX, FSS) and the relative game score as measure of participants' performance (see Section 2.3). Further, the relative difference between the counted and real number of oddballs was used as performance measure for the secondary task. *p*‐values were corrected according to Greenhouse–Geisser. Post hoc analysis consisted of multiple pairwise comparisons with Tukey's HSD (honestly significant difference) test. The significance threshold was set to *α* < 0.05 after correcting for family‐wise error rate.

#### 2.5.2 Processing of the EEG Data

The EEG signals were de‐trended and bandpass filtered using a fourth‐order infinite impulse response Butterworth filter with the cutoff frequencies 0.2 and 20.0 Hz. Afterward, signals were segmented into epochs of 1 s using the onset of the sound of interest. A 200‐ms baseline interval before the onset was included. The FASTER pipeline of Nolan et al. (2010), incorporating independent component analysis (ICA) as implemented in mne‐Python version 1.6.1 (Gramfort et al. 2014), was applied to the epoched data to remove both physiological (cardiac, ocular, and muscle‐related) and nonphysiological (power interference) artifacts (Chaumon et al. 2015; Hipp and Siegel 2013; Lee et al. 1999). Only channels and epochs that passed a threshold‐based rejection (threshold channels: 5 standard deviations; SD; threshold epochs: 3 SD) were used in the ICA and further analysis. ICA was performed automatically using the EOG and ECG channels for correlation‐based artifact detection, signal kurtosis for high‐amplitude offsets, Hurst exponent for nonbiological artifacts, power gradient for residual white noise, and median gradient for muscle‐related artifacts (Nolan et al. 2010). After cleaning the signals, epochs were baseline corrected by subtracting the mean amplitude of the time interval before the sound onset (200 ms), and bad channels were interpolated per epoch using spline interpolation (Gramfort et al. 2014; Nolan et al. 2010). Finally, the scalp sensor signals were processed using current source density (CSD) analysis, estimated through the surface Laplacian method. This reference‐independent approach enhances the spatial resolution of the data compared to traditional scalp potential analysis (Kayser and Tenke 2015; Yao et al. 2019).

##### 2.5.2.1 Mass‐Univariate Analysis of Event‐Related Potentials

To identify differences in ERPs between the experimental conditions in a univariate group‐level statistic, one‐sided nonparametric permutation‐based clustering (Maris and Oostenveld 2007) with a rmANOVA and the two factors *difficulty level* and *ergonomic position* was performed. Post hoc testing was performed using a two‐sided *t*‐test permutation‐based clustering per contrast of interest. Clusters were identified as adjacent EEG channels and time points using a cluster‐level and group‐level threshold of *α* < 0.05.

##### 2.5.2.2 Multivariate Pattern Analysis of Event‐Related Potentials

In the final analysis, we enhanced the sensitivity to distinguish between the conditions, thereby increasing the statistical power (Holdgraf et al. 2017; Kriegeskorte and Douglas 2019), by employing a subject‐wise multivariate pattern analysis (MVPA). MVPA is particularly powerful for investigating how the brain encodes information, as it captures the multidimensional nature of neurophysiological data and effectively addresses the interindividual variability in both anatomical and functional brain patterns (Marsicano et al. 2024). For this analysis, the epoched data were downsampled to 100 Hz, and the conditions to be decoded (referred to as classes in the MVPA) were divided into training and testing sets. A fivefold stratified repeated cross‐validation with 20 iterations was performed, resulting in a total of 100 folds per time point.

To allow plausible neurophysiological interpretation of the decoded patterns, we chose a linear model for supervised machine learning. This choice allows for inverse computations of the model coefficients by transforming the decoding weights back into activation patterns, which makes it possible to interpret how the brain encodes information (Haufe et al. 2014). We selected a linear discriminant analysis (LDA) with the least squares solution as a solver and an automatic shrinkage using the Ledoit–Wolf lemma (as implemented in scikit‐learn version 1.4.1). A dummy classifier with a stratified classification strategy estimated an empirical baseline. The decoding was conducted on a time point–by–time point basis using the sliding estimator from mne‐Python version 1.6.1 (Gramfort et al. 2014).

The area under the receiver operating characteristic curve (AUC) quantified accuracy for the two‐class classification (one for each contrast), while a weighted F1 score was applied for the three‐class classification. Decoding performance was statistically evaluated by bootstrapping the classification scores of the folds in a Monte Carlo simulation (5000 iterations) and computing the bootstrapped mean and its 95% confidence interval (CI; Cumming 2014). Classification time intervals were considered significant if the lower CI of the LDA performance exceeded the upper CI of the dummy classifier for at least 200 consecutive milliseconds.

The decoding coefficients of the classification models were transformed into interpretable patterns (Haufe et al. 2014), averaged across participants, and visualized on topographic maps. To ensure that patterns were meaningful and generalized across participants, we applied a spatiotemporal mask using univariate bootstrapped means and CIs (5000 iterations). Only patterns at electrode positions where the evoked response to the oddball sounds of one condition differed from the other two conditions were visualized. The visualized patterns provide information on how the values in the sensors contribute to the prediction of a class label. When interpreting the patterns, it should be noted that the pattern values reflect the contribution to the classification, rather than the direction and strength of the underlying evoked responses (Haufe et al. 2014). For the binary decoding patterns, positive values indicate that the particular region contributes to the selection of the second class, whereas negative values at a particular location are less informative. In a three‐class classification, positive pattern values indicate a higher probability that a data sample will be classified in the target class, while negative pattern values decrease this probability, suggesting that activation in the respective sensors is more likely to be associated with the remaining classes. The closer the assigned patterns are to zero, which represents the decision boundary of the classifier, the less confident the model is in its prediction. To examine the relationship between evoked response amplitudes and significant patterns, bootstrapped means and their CIs of the conditions in each significant pattern electrode region were extracted for the time point of maximal classification performance.

## 3 Results

### 3.1 Behavioral and Subjective Results

**FIGURE 3:**
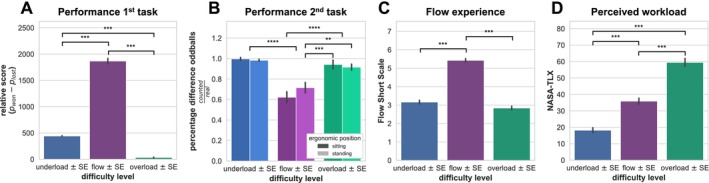
Task performance measures and subjective experiences. *Note:* Group means of (A) relative game score, (B) difference of counted oddballs and real number for each ergonomic position, (C) FSS, and (D) NASA‐TLX are plotted for each difficulty level in the primary task. Black lines indicate standard errors. Significant differences are indicated with asterisks (**p ≤ 0.01, ***p ≤ 0.001, ****p ≤ 0.0001). Abbreviations: *p*_lost_, points lost during the game; *p*_won_, points won during the game; SE, standard error.

A significant main effect of difficulty level on the relative score (i.e., performance in the primary task; *F*(2,24) = 241.14, *p* < 0.001, $\eta_{p}^{2}$ = 0.95) was found (see Figure 3). This effect was significant between all conditions (for details, see Table A2 and Table A3). No significant main or interaction effects of ergonomic position were observed. A significant interaction was found for the number of counted oddballs relative to the correct number (i.e., performance in the secondary task; *F*(2,24) = 3.67, *p* < 0.05, $\eta_{p}^{2}$ = 0.23). Tukey's HSD showed that highly significant differences were found between both underload and overload conditions compared with flow conditions, respectively. Specifically, standing had a positive effect on performance in the secondary task during the flow condition, while sitting had a positive effect on task performance during underload and overload. The pairwise comparisons show that the flow conditions (both sitting and standing) are associated with worse performance in the secondary task compared to both overload and underload conditions. There were no significant differences between overload and underload conditions, or between the sitting and standing conditions within overload and underload groups (see Figure 3; for details, see Table A6 and Table A7).

For the subjective measures, a significant main effect of difficulty level on the NASA‐TLX was found (*F*(2,24) = 52.78, *p* < 0.001,$\;\eta_{p}^{2}$ = 0.85). Pairwise comparisons showed significant differences between all workload conditions for the NASA‐TLX score (see Figure 3; for details, see Table A8 and Table A9). Finally, the rmANOVA for the FSS also showed a significant main effect of difficulty level (*F*(2,24) = 66.78, *p* < 0.001,$\;\eta_{p}^{2}$ = 0.85). Tukey's HSD showed that, for FSS, only the difference between the flow condition and the other conditions was significant (for details, see Table A6 and Table A7).

### 3.2 Mass‐Univariate Analysis of Event‐Related Potentials

**FIGURE 4:**
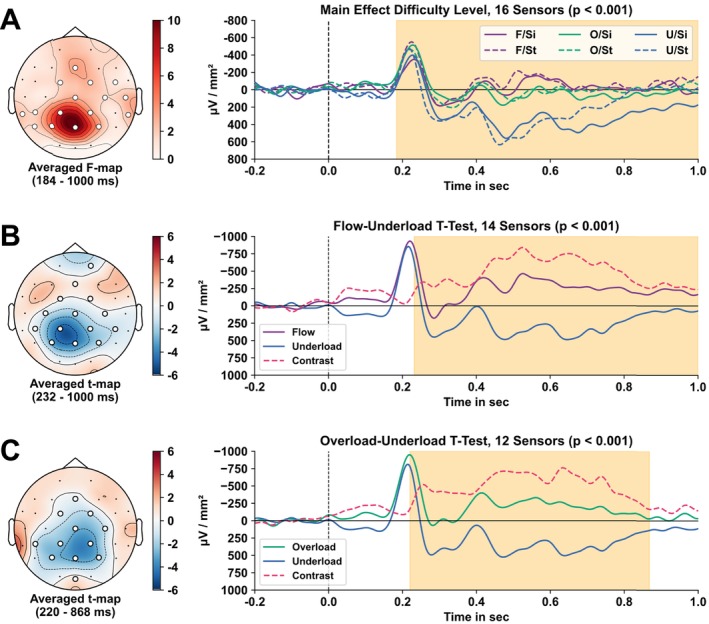
Permutation‐based spatiotemporal clusters of the auditory oddball ERPs in the rmANOVA and pairwise comparisons. *Note:* (A) Spatiotemporal *F*‐test cluster for the main effect difficulty level with signals corresponding to auditory ERPs of each difficulty level and ergonomic position, averaged over significant electrodes. Spatiotemporal‐dependent *t*‐test cluster for pairwise comparisons of auditory ERPs corresponding to the (B) flow and underload condition and (C) overload and underload condition, respectively. The color bar indicates the magnitude of the test statistic. Electrodes of significant clusters are marked with white circles. The left panels show the topographic map of the test statistics with *F*‐values and *t*‐values averaged over a significant time window after stimulus onset. The right panels display averaged signals over significant electrodes per condition and corresponding contrast over time after stimulus onset (time point zero). The orange highlighted area indicates a significant difference between conditions in this time window. Abbreviations: *F*, flow; *O*, overload; *U*, underload; Si, sitting; St, standing.

In the permutation‐based cluster analysis, we found a significant main effect of difficulty level (*p*_F‐statistic_ ≤ 0.001) on ERPs locked to the onset of the oddball sound. The significant spatiotemporal cluster covering 16 electrodes over centroparietal regions emerged after 184 ms and lasted until the end of the analysis time interval (1000 ms; see Figure 4A). We did not observe a significant effect of the ergonomic position or the two main effects interacting.

The post hoc *t*‐statistic clustering revealed a significant difference between the conditions flow and underload (*p*_*t*‐statistic_ < 0.001) as well as overload and underload (*p*_*t*‐statistic_ < 0.001). The spatiotemporal cluster discriminating between flow and underload comprised 14 sensors located over centroparietal regions. ERPs in these sensors started to differ after 232 ms with overall more negative values during the flow compared to underload condition. The cluster lasted until 1000 ms after sound onset. The second significant contrast overload–underload revealed a spatiotemporal cluster that started at 220 ms and lasted until 868 ms after sound onset. It showed a significant difference in the evoked potentials with more negative values in the overload condition within the time intervals in 12 electrodes located over parietal, central, and centrofrontal regions, similar to the flow–underload cluster. No significant clusters were found when contrasting flow and overload, indicating no differences in the evoked potentials between conditions in the mass‐univariate cluster analysis.

### 3.3 Multivariate Pattern Analysis of Event‐Related Potentials

**FIGURE 5:**
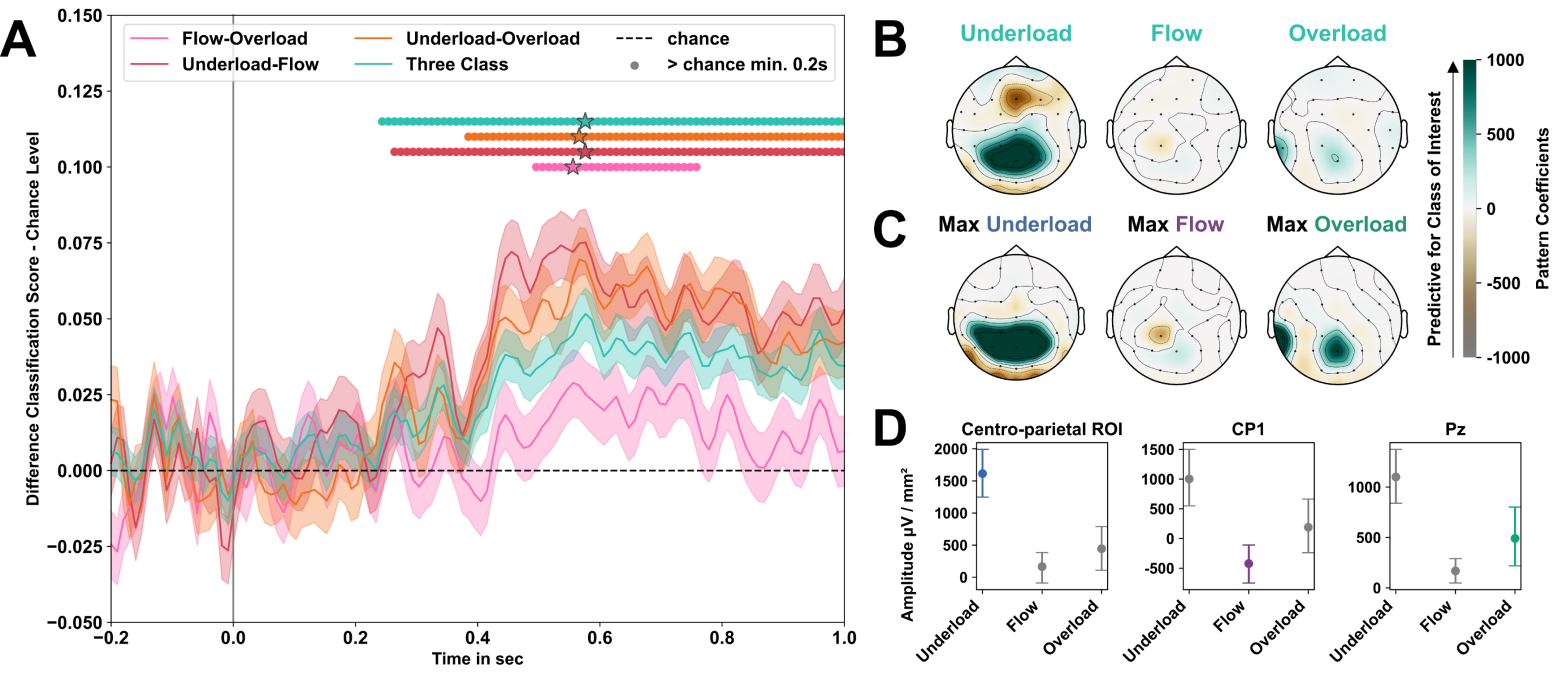
Classification results for the binary (contrasts) and three‐class MVPA‐based LDA. *Note:* (A) Average LDA classification performance and respective confidence intervals (CI) across folds and subjects in relation to the estimated chance level (upper CI of the dummy classifier performance) for the time interval of the oddball ERP. (B) Averaged and (C) maximum pattern coefficients of the significant time intervals for three‐class decoding extracted from the trained LDA models. (D) Evoked responses per condition in the electrodes of the pattern regions. Classification intervals were deemed significant if the lower CI of the LDA performance exceeded the upper CI of the dummy classifier for a minimum of 200 consecutive milliseconds. Significant time points are highlighted with colored circles. Star icon indicates the peak (max) of the classification score across all significant time points. Positive pattern values increase the likelihood of classifying the observed condition, while negative values in spatial patterns decrease this likelihood. Centroparietal region of interest includes electrodes at the positions CP1, CP2, P3, Pz, and P4.

Results of the temporal decoding are summarized in Table 1 and Figure 5. The decoding contrast underload–flow, and the three‐class decoding began to show above‐chance level classification between conditions approximately 250 ms after the onset of the oddball stimulus. This was followed by the underload–overload, and finally, flow–overload contrast (see Table 1). For most contrasts, classification remained above chance level until the end of the 1‐s analysis window, except for the flow–overload contrast. We were able to significantly distinguish flow states from overload only during a narrow time window of approximately 260 ms (496‐ to 758‐ms post‐stimulus onset). The peak classification scores across all decoding contrasts were observed within a similar time frame, ranging between 556 and 576 ms after stimulus onset. The highest classification accuracy was achieved for decoding underload and flow, with a peak AUC score of 57.5% CI (56.45; 58.59] and mean AUC score of 55.12% CI (54.03; 56.19). In contrast, the lowest classification accuracy was found in the flow–overload decoding, with a peak AUC score of 53.17% CI (52.04; 54.26) and mean AUC score of 52.51% CI (51.4; 53.61). Comparing mean classification scores and CIs relative to the chance level (upper CI of the dummy classifier) over the significant time window, we observed significantly lower performance for the three‐class (Diff: 3.32% CI [2.52; 4.12]) and flow–overload (Diff: 2.23% CI [1.12; 3.33]) decoding compared to both the underload–flow (Diff: 5.14% [4.06; 6.21]) and underload–overload (Diff: 5.03% [3.93; 6.11]) decodings. There was no difference in the performance between the three‐class and flow–overload decodings, as well as the underload–flow and underload–overload decoding (see Figure 5A).

**TABLE 1:** Statistics of the LDA classification scores as well as dummy classifier per decoding contrast*.*

Decoding contrast	Time range [duration] (ms)	Max score [%CI]	Time max (ms)	Mean score [%CI]	SD score [%CI]	Dummy [%CI]
Underload—flow	263–1000 [737]	57.5 [56.45; 58.59]	576	55.12 [54.03; 56.19]	1.48 [1.49; 1.47]	49.84 [49.71; 49.98]
Underload—overload	384–1000 [616]	56.62 [55.49; 57.67]	566	54.7 [53.59; 55.78]	1.09 [1.1; 1.09]	49.49 [49.31; 49.67]
Flow—overload	495–758 [262]	53.17 [52.04; 54.26]	556	52.51 [51.4; 53.61]	0.48 [0.47; 0.48]	50.17 [50.05; 50.28]
Three class	243–1000 [757]	37.94 [37.07; 38.79]	576	36.09 [35.3; 36.89]	1.05 [1.04; 1.06]	31.9 [31.07; 32.77]

*Note:* Time range with start, end, and duration in milliseconds of the significant time interval, as well as maximum (Max), mean, and standard deviation (SD) of the linear discriminant analysis (LDA) classification score during the significant time interval are shown for each decoding contrast. Classification intervals were significant if the lower CI of the mean LDA performance exceeded the upper CI of the dummy classifier for a minimum of 200 consecutive milliseconds.

#### 3.3.1 Spatial Distribution of the Patterns From the Temporal Decoding

**FIGURE A4:**
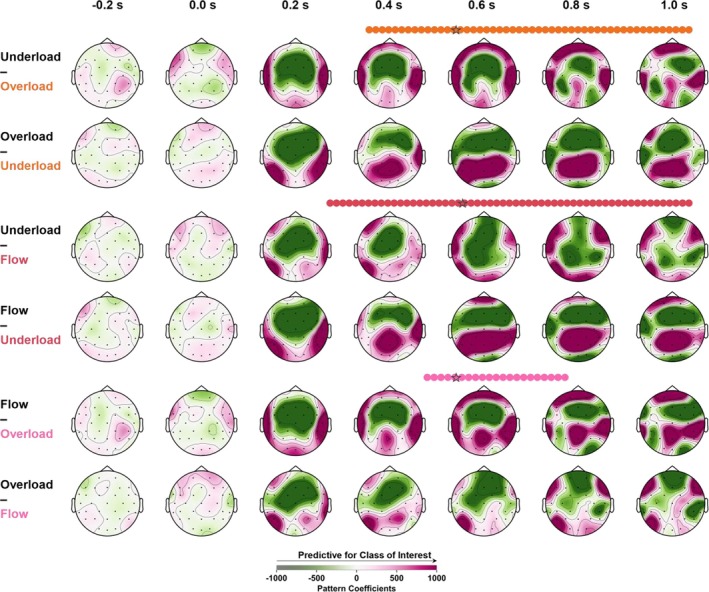
Spatial patterns of binary MVPA classification over ERP time interval*. Note:* Spatial patterns of binary classification coefficients for the ERP time interval of the auditory oddball sound. In binary classification, positive values increase the likelihood of classifying the observed condition as the second class (highlighted in color), while negative values in spatial patterns decrease this likelihood. Classification intervals were deemed significant if the lower CI of the LDA performance exceeded the upper CI of the dummy classifier for a minimum of 200 consecutive milliseconds. Significant time points are highlighted with colored circles. Star icons indicate the maximum of the classification score across all time points.

**FIGURE A5:**
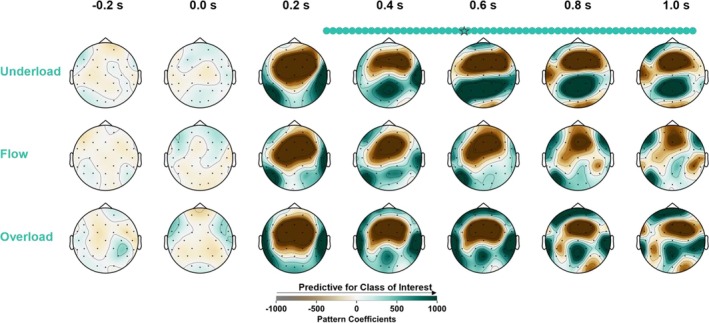
Spatial patterns of three‐class MVPA classification over ERP time interval. *Note:* Spatial patterns of three‐class classification coefficients for the ERP time interval of the auditory oddball sound. In three‐class classification, positive values increase the likelihood of classifying the observed condition (labeled per row), while negative values in spatial patterns decrease this likelihood. Classification intervals were deemed significant if the lower CI of the LDA performance exceeded the upper CI of the dummy classifier for a minimum of 200 consecutive milliseconds. Significant time points are highlighted with colored circles. A star icon indicates the maximum of the classification score across all time points.

Interpretable coefficient patterns were averaged across participants and visualized on topographic maps (see Figure 5). The temporal evolution of the decoding patterns over the analyzed time window is provided in the Appendix in Figure A4 for the binary decoding and Figure A5 for the three‐class decoding. The three‐class decoding allowed us to derive class‐specific patterns when comparing the class of interest to all remaining classes. Figure 5B shows the significant patterns per class of the three‐class decoding averaged across participants and the significant decoding time interval. Figure 5C depicts the significant decoding patterns per class at the time point of maximum classification performance averaged across participants. Predictive patterns were observed in electrodes positioned over the centroparietal, midfrontal, and left temporoparietal regions. Figure 5B and 5C illustrate that activation patterns in electrodes located over parietocentral regions were most indicative of the underload condition. A negative frontal pattern showed that activation in the Fz electrode was more associated with the other two classes. Activation patterns over the left temporoparietal region (TP9), as well as over a very focal parietal area, (Pz) contributed to the choice of the overload condition. Decoding of flow states was visible by the absence of activation patterns at electrodes overlying parietal regions, localized around the CP1 electrode. Evoked responses in the parietal electrodes, identified as the region of interest for decoding, showed the strongest positive amplitudes during underload (centroparietal sensors), followed by overload (Pz), with the smallest deflections occurring during flow (CP1) at the time of peak classification performance (see Figure 5D).

## 4 Discussion

The current study investigated how varying levels of mental workload influence attentional allocation and auditory evoked brain responses, with a particular focus on the flow state immersion and a flow‐persevering secondary oddball dual‐task paradigm. In flow research, studies have yet to provide a comprehensive understanding of the neural signatures and attentional resources during flow experiences (e.g., Kotler et al. 2022; van der Linden et al. 2021), particularly in dual‐task scenarios. To address this research question, we induced the experience of flow in a dual‐task paradigm with an implicit secondary task response and varying levels of mental workload (underload, flow, and overload) using EEG. Participants engaged in a primary game task while simultaneously performing an auditory implicit (silent counting) oddball task, which allowed for assessing their available attentional resources. By combining subjective, electrophysiological, and behavioral measures, we identified distinct differences in attention‐related ERPs between flow, underload, and overload. Furthermore, the subjective and behavioral measures confirmed the experience of flow, including a positive valence and optimal performance, when the skill‐challenge balance was tailored to the participant (i.e., the flow condition). We further explored whether ergonomic positions commonly implemented in office workplaces (i.e., standing and sitting) affect the experience of flow and allocation of attention.

### 4.1 Modulated P300 Indicates Attention Allocation, Unspecific to Flow

First, we examined how varying workload levels and ergonomic positions influenced ERPs elicited by the oddball sound in the secondary task using a mass‐univariate group‐level analysis (Maris and Oostenveld 2007). Our findings confirmed the hypothesis that attention to the secondary task was modulated by the difficulty of the primary game task. The ergonomic position had no measurable effect on the event‐related responses. Specifically, we observed significant differences in current densities during the flow and overload conditions compared to underload, with reduced centroparietal CSD amplitudes likely reflecting the P300 component (see Figure 4). These findings of a decreased parietal P300 amplitude are in line with prior dual‐task studies (Núñez Castellar et al. 2019). Notably, reduced P300 amplitudes were observed during both overload and flow, with no significant differences between conditions (see also Núñez Castellar et al. 2019 for similar results). Attenuation of P300 has been shown to be modulated by states of high workload (Maclin et al. 2011; Núñez Castellar et al. 2019; Polich 2007). Our spatiotemporal clustering results suggest that both flow and overload experiences prioritize the primary game task, with less attentional reallocation to the implicit secondary task. This alignment of electrophysiological responses for flow and overload supports the theory that flow involves resource‐intensive attention processes much like high‐effort scenarios (Alameda et al. 2022). Contrary to our hypothesis, we found no significant spatiotemporal cluster between flow and overload in the mass‐univariate analysis, despite participants subjectively reporting differences in workload, as well as increased enjoyment and reward associated with flow (see Figure 3).

In conclusion, our findings suggest that the attenuation of the P300 component in response to the secondary task stimulus reflects reduced attention allocation. This phenomenon may be attributed to the highly attentionally demanding primary task. Importantly, the group‐level statistical approach of a mass‐univariate clustering could not differentiate flow and overload and identify the distinct flow signature of focused attention.

### 4.2 Temporal Decoding of Flow States With Multivariate Pattern Analysis

Compared to mass‐univariate clustering, the data‐driven approach of MVPA offers key advantages by (1) accounting for variability in the origins of electrophysiological signatures across subjects and (2) investigating activation patterns in a multidimensional plane, thereby increasing statistical sensitivity (Holdgraf et al. 2017; Kriegeskorte and Douglas 2019; Marsicano et al. 2024). Consequently, it is particularly suited for investigating flow experiences, which likely involve complex neurocognitive dynamics and individual‐specific processing strategies (Marsicano et al. 2024).

Thus, to identify the flow‐specific neuronal signature and differentiate between all three workload conditions, we employed a subject‐wise MVPA. By applying MVPA, we were able to reveal significant differences in neuronal responses to the auditory oddballs between flow and both underload and overload conditions, advancing our understanding of how these states are encoded in the brain. Aligned with our mass‐univariate analysis, the MVPA results further corroborated that flow and overload states are the most difficult to distinguish, particularly when examining modulations in auditory ERPs. This was evidenced by the lowest mean accuracy and shortest time interval of above‐chance‐level classification (see Table 1 and Figure 5A).

Examining the temporal evolution of the binary and three‐class decoding, above chance level classifications started approximately 250 ms after the oddball sound onsets and lasted until the end of the analysis time window in most decoding contrasts (see Figure 5A, as well as Figure A4 and Figure A5). The time intervals that allowed significant classifications between conditions were similar to the spatiotemporal clusters identified in the mass‐univariate analysis. Notably, the significant distinction of classes was achieved through late components of auditory ERPs, likely reflecting top‐down, rather than bottom‐up, processes related to the dual‐task paradigm (Debener et al. 2002; Polich 2007).

In line with our results in the spatiotemporal cluster analysis, the P300 component elicited by the oddball sound was identified as the main region of interest for the decoding and allowed to discriminate the different states (see Figure 5A). Contrary to the mass‐univariate analysis, we observed a stepwise differentiation between all three conditions. Modulation of activity in electrodes overlying centroparietal regions was highest during underload, followed by overload, and was almost absent during the experience of flow (see Figure 5B–D). Taken together, the spatiotemporal clusters and MVPA decoding patterns provide convergent evidence that the experience of flow functions similarly to a shielding mechanism. These results suggest that during flow attention is most efficiently focused on the primary task and shielded from less prioritized stimuli, such as the auditory oddball in the secondary task.

**FIGURE A6:**
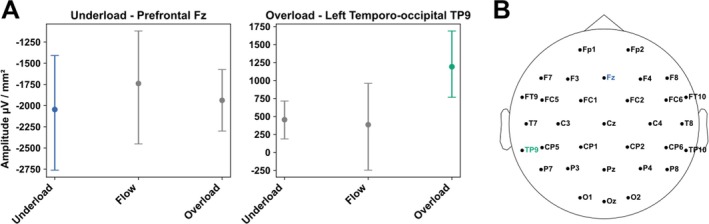
Analysis of the regions of interest derived from the spatial patterns of the three‐class MVPA classification at the time point of maximum performance.

Contrary to previous findings (Núñez Castellar et al. 2019), activation patterns in frontocentral regions did not allow differentiation between the three states in our study (see Figure A6 and Table A1). Instead, our results suggest that activity in these regions is linked to top‐down modulation and attentional control (Herrmann and Knight 2001), which are similarly engaged in all three conditions. This top‐down prefrontal control is likely necessary to perform the subprocesses associated with an overt response to a secondary task, which include task switching, attention allocation, stimulus processing, working memory information updating, and response selection.

Finally, activation patterns in electrodes overlying temporoparietal regions contributed to the choice of the overload class (see Figure 5B,C, as well as Figure A5 and Figure A6). These higher evoked potentials in response to the oddball sound during overload may be explained by disengagement from the primary task due to its high difficulty level and a shift of attention toward the secondary task. This interpretation is further corroborated by behavioral results. Overload resulted in significantly worse performance in the primary task compared to both flow and underload. However, pairwise comparisons indicated that performance in the secondary task was not different in overload compared to underload (see Figure 3).

### 4.3 Behavioral and Subjective Results Support Flow Experience Manipulation

Subjective questionnaire results confirmed that participants experienced flow during the respective condition of the primary task and varying levels of mental load during all difficulty levels (see Figure 3C,D). Furthermore, experiencing flow correlated with the highest performance in the primary task, albeit not the highest subjective workload. This was expected as the flow experience is associated with an immersed focus on task‐relevant stimuli and feelings of enjoyment during task performance. Notably, we observed an inverted U‐shape in our performance measure (see Figure 3A) paralleling reported arousal measures during flow (Peifer et al. 2014; Ulrich et al. 2016) and reported subjective flow experience (see Figure 3C and Alameda et al. 2022).

### 4.4 Ergonomic Position Affects Secondary Task Only

We did not find significant differences between ergonomic positions in the primary task or subjective measures. Regarding the secondary task, a significant interaction was found between the difficulty level in the primary task and ergonomic position for the performance measure, i.e., the difference between counted oddballs and the correct number. Specifically, performance was positively affected by the standing position in the flow condition, whereas the opposite effect was shown for underload and overload (see Figure 3B). This interaction indicates that standing has a positive effect on the secondary task performance during flow. Consistent with our findings, previous studies have reported positive effects of standing on task engagement (Finch et al. 2017) and mouse behavior during computer desk work (Ghesmaty Sangachin et al. 2016). However, it is notable that the participants in the latter study also perceived the standing condition to be more demanding in terms of the workload (Ghesmaty Sangachin et al. 2016). To conclude, the ergonomic position affected only the behavioral performance in the secondary task. Since we did not investigate individual preferences for an ergonomic position, future studies could explore whether the preferred ergonomic position influences behavioral performance in both tasks, as well as the electrophysiological responses in the dual task.

### 4.5 Flow as Attention‐Shielding Mechanism

Integrating the findings from electrophysiological and behavioral responses, we identified an attention‐shielding mechanism during the experience of flow. This was evident in the absence of evoked responses in the parietal regions to the auditory stimuli of the secondary task. Although similar evoked responses were observed for flow and overload in the spatiotemporal clustering, the MVPA successfully discriminated between the two conditions based on the involvement of activity in parietal regions. We conclude that, in a state of flow, top‐down control processes strongly prioritize the primary task, effectively shielding it from other stimuli. This shielding effect facilitated the phenomenon of being fully tuned in. Our behavioral and subjective findings confirmed that flow was perceived as less effortful than overload and as highly pleasurable, enabling high performance with an experience of subjective pleasantness and positive valence.

### 4.6 Limitations and Future Work

The current study identified spatiotemporal correlates of attention and immersion linked to the flow experience within a dual‐task paradigm. We focused on ERPs and conceptualized flow as a holistic, positive experience of deep absorption during gaming at the optimal skill‐challenge balance. The emergence of flow was facilitated through tailoring the difficulty level to each participant's skill and validated through established questionnaires assessing the subjective experience of flow (Rheinberg et al. 2003). However, it is important to note that flow is defined as a multidimensional construct (Csikszentmihalyi 1975). To understand the relevance of each dimension of flow to its emergence and neurophysiological signatures, research studies need to specifically target and systematically manipulate this dimension. Hence, future studies are necessary to explore the role of dimensions beyond the skill‐challenge balance and immersion, such as autotelicity or sense of time perception (Durcan et al. 2024). In this context, spontaneous oscillatory power analyses allow investigating neural information coding, attentional modulation, and interareal communication during flow (van der Linden et al. 2021). This is particularly relevant in the absence of environmental stimuli related to a secondary task. Furthermore, oscillatory power measures indexing workload (Gevins et al. 1995), engagement (Pope et al. 1995), and emotion processing (Smith et al. 2017) can be investigated. Another limitation of the presented study is the relatively small sample size, potentially affecting the sensitivity to some effects, particularly regarding ergonomic positions. Finally, while our primary focus was on the interpretability and electrophysiological insights gained from the MVPA, a key next step is to optimize the machine learning pipeline, including feature engineering, to enhance classification performance in brain–computer interface (BCI) applications for detecting flow states.

### 4.7 Implications

This study has significant implications for the implicit identification of flow states using EEG, which can be applied in BCI technologies for real‐world scenarios. By leveraging implicit flow monitoring, it is possible to provide real‐time feedback when the flow state is reached, without interrupting the individual's experience. Additionally, this approach supports the fostering of flow‐inducing factors in environments by continuously monitoring both the flow state and relevant variables. Importantly, flow can be achieved without the subjective experience of overload, characterized by perceived high workload, physical stress to the limit of exhaustion, and reduced positive valence. Understanding this protective function holds considerable promise for educational design, workplace productivity, and mental health, offering opportunities to create environments that promote cognitive functioning and well‐being. Our findings demonstrate that flow can be distinguished through evoked potentials, enabling individuals to manage attention and working memory resources effectively without constantly reacting to external stimuli.

## 5 Conclusion

By developing a flow‐inducing game‐based dual‐task paradigm and assessing auditory attention allocation using ERPs, we measured flow experience implicitly without interfering with the task. To our knowledge, this study is the first to apply MVPA to differentiate flow from other states of mental workload. By identifying an activation pattern specific to flow, the work represents a significant milestone in the research on the electrophysiological basis of flow. We successfully distinguished the flow state from states of cognitive overload and underload based on EEG measures alone. Supported by subjective and behavioral results, our EEG‐based measurements highlight that the experience of flow is an attention‐focusing mechanism associated with positive valence and greatly enhanced performance compared to other workload conditions. The results contribute to the holistic understanding of flow, and our complementary approach pioneers the study of mental states characterized by strong interindividual variations such as flow. The implications of implicitly evaluating flow experiences are particularly relevant for BCIs in applied contexts that benefit from maintaining high attentional focus, performance, and perceived low cognitive load.

## Author Contributions

**Katharina Lingelbach:** formal analysis, data curation, supervision, validation, visualization, writing – original draft preparation, writing – review and editing. **Anna Vorreuther:** formal analysis, data curation, validation, visualization, writing – original draft preparation, writing – review and editing. **Elias Moll:** investigation, methodology. **Mathias Vukelić:** conceptualization, funding acquisition, methodology, project administration, supervision, writing – review and editing.

## Conflicts of Interest

The authors declare no conflicts of interest.

## Peer Review

The peer review history for this article is available at https://www.webofscience.com/api/gateway/wos/peer‐review/10.1111/ejn.70283.

## Figures and Tables

**Figure:**
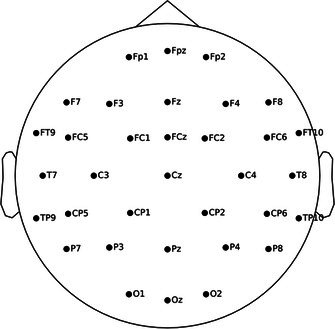
FIGURE A1 Electrode layout*. Note:* Electrodes were placed according to 10–20 system (32 electrodes). Common reference was set to FCz, ground electrode was set to Fpz.

**Figure:**
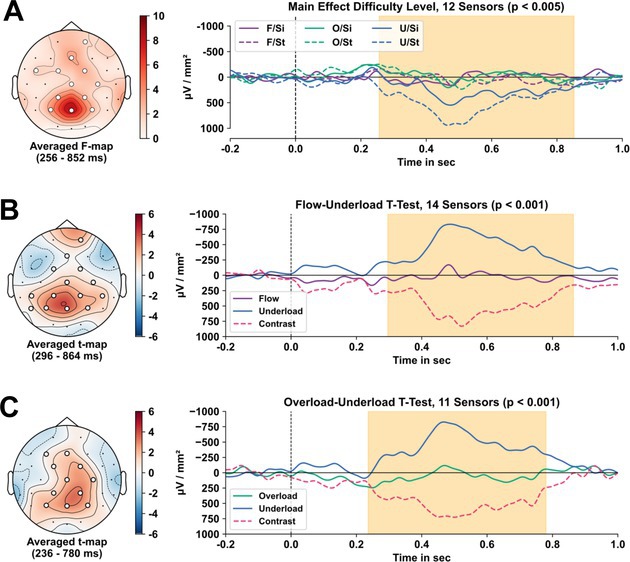
FIGURE A2 Difference of oddball and frequent sound ERP clusters per condition. *Note:* Results of the spatiotemporal cluster analyses of difference between auditory oddball and frequent sound ERPs for all conditions. The left panels show the topographical map of test statistics (*F*‐values and *t*‐values, respectively), averaged over a significant time window after stimulus onset. The color bar indicates the magnitude of the test statistics. Electrodes of significant clusters are marked with white circles. The right panels display averaged signals per condition and corresponding contrast over time after stimulus onset (time point zero). The highlighted orange area indicates a significant difference between conditions in this time window. (A) Spatiotemporal *F* test cluster corresponding to auditory ERPs of each difficulty level and ergonomic position. A cluster of 12 sensors was found in a time window ranging from approx. 256 to 852 ms (p<0.005). (B) Spatiotemporal *t* value cluster for pairwise comparisons of auditory ERPs corresponding to flow and underload condition, respectively. A cluster of 14 sensors was found in a time window ranging from approx. 300 to 900 ms (p<0.001). (C) Spatiotemporal *t* value cluster for pairwise comparisons of auditory ERPs corresponding to overload and underload condition, respectively. A cluster of 11 sensors was found in a time window ranging from approx. 200 to 800 ms (p<0.001). Abbreviations: F: flow; O: overload; Si: sitting; St: standing; U: underload.

**Figure:**
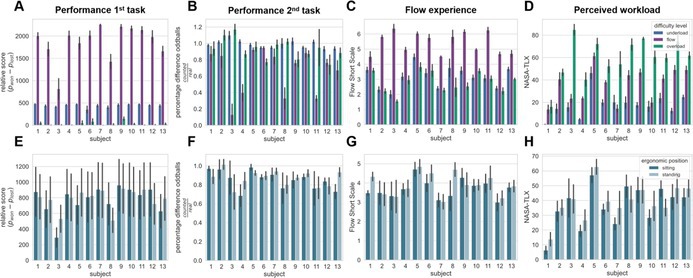
FIGURE A3 Performance and subjective experiences of primary task per subject. *Note:* Subject‐wise means of (A) relative game score, (B) difference of counted oddballs and real number for each ergonomic position, (C) FSS, and (D) NASA‐TLX per difficulty level in the primary task and subject‐wise means of (E) relative game score, (F) difference of counted oddballs and real number for each ergonomic position, (G) FSS, and (H) NASA‐TLX per ergonomic position. Black lines indicate standard errors. Abbreviations: $p_{\text{lost}}$: points lost during the game; $p_{\mathrm{won}}$: points won during the game.

## Data Availability

Code and data pertaining to this study are available under the associated Open Science Framework Project https://osf.io/vfcjd/.

## Appendix A

**TABLE A1:** Statistics of three‐class MVPA classification.

	Flow–underload	Overload–underload	Overload–flow
Prefrontal—Fz	307.1 [−665.7; 1153.4]	108.2 [−663.3; 872.5]	−198.8 [−803.6; 415.7]
Centroparietal sensors	**−1448.2** **[−2074; −898.2]**	**−1168.6** **[−1712.3; −613.8]**	279.6 [−216.4; 832.1]
Centroparietal CP1	**−1426** **[−1936.9; −917]**	**−813.6** **[−1482.6; −122.1]**	**612.3** **[92.9; 1112.1]**
Parietal Pz	**−1863.9** **[−2631.6; −1149.8]**	**−1219.5** **[−2064.2; −470.6]**	**644.4** **[20.7; 1389.2]**
Left temporo‐occipital TP9	−69.9 [−843.6; 591.2]	**736.7** **[88.4; 1466.7]**	806.6 [−83.3; 2022.1]

*Note:* Bootstrapped means and confidence intervals of the evoked potentials per contrasts for each region of interest derived from the spatial patterns of the three‐class MVPA classification at the time point of maximum performance. Significant comparisons are highlighted in bold.

**TABLE A2:** rmANOVA results for first task performance: relative score*.*

Variable	SS	df [1,2]	MS	*F*	*p*	*p* (GG)	$\eta_{p}^{2}$	*ε*
Difficulty level	482,128 25.926	[2,24]	24106412.963	241.139	< 0.001	< 0.001	0.953	0.517
Ergonomic position	3784.758	[1,12]	3784.758	0.143	0.712	0.712	0.012	1
Difficulty level * ergonomic position	680.627	[2,24]	340.313	0.013	0.988	0.93	0.001	0.557

Abbreviation: GG, Greenhouse–Geisser.

**TABLE A3:** Tukey's HSD results for first task performance: relative score.

Condition 1	Condition 2	Mean difference	CI	*p*
Flow	Overload	−1834.231	[−1938.8488, −1729.6127]	< 0.001
Flow	Underload	−1425.256	[−1529.8744, −1320.6384]	< 0.001
Overload	Underload	408.974	[304.3563, 513.5924]	< 0.001

**Table:** TABLE A4 rmANOVA results for second task performance: difference of counted and real number of oddball sounds.

Variable	SS	df [1,2]	MS	F	*p*	*p* (GG)	$\eta_{p}^{2}$	*ε*
Difficulty level	1.511	[2,24]	0.756	9.968	0.001	0.006	0.454	0.559
Ergonomic position	0.006	[1,12]	0.006	0.461	0.51	0.51	0.037	1
Difficulty level * ergonomic position	0.056	[2,24]	0.028	3.671	0.041	0.05	0.234	0.847

Abbreviation: GG, Greenhouse–Geisser.

**Table:** TABLE A5 Tukey's HSD results for second task performance: difference of counted and real number of oddball sounds.

Condition 1	Condition 2	Mean difference	CI	*p*
Flow * sitting	Flow * standing	0.094	[−0.0618, 0.2488]	0.5133
Flow * sitting	Overload * sitting	0.320	[0.1645, 0.4751]	< 0.001
Flow * sitting	Overload * standing	0.293	[0.1381, 0.4487]	< 0.001
Flow * sitting	Underload * sitting	0.3747	[0.2194, 0.53]	< 0.001
Flow * sitting	Underload * standing	0.361	[0.2057, 0.5163]	< 0.001
Flow * standing	Overload * sitting	0.2263	[0.071, 0.3816]	0.0006
Flow * standing	Overload * standing	0.1999	[0.0446, 0.3552]	0.0036
Flow * standing	Underload * sitting	0.2812	[0.1259, 0.4365]	< 0.001
Flow * standing	Underload * standing	0.2675	[0.1122, 0.4228]	< 0.001
Overload * sitting	Overload * standing	−0.0264	[−0.1817, 0.1289]	0.9965
Overload * sitting	Underload * sitting	0.0549	[−0.1004, 0.2102]	0.9123
Overload * sitting	Underload * standing	0.0412	[−0.1141, 0.1965]	0.9734
Overload * standing	Underload * sitting	0.0813	[−0.074, 0.2366]	0.6614
Overload * standing	Underload * standing	0.0676	[−0.0877, 0.2229]	0.8107
Underload * sitting	Underload * standing	−0.0137	[−0.169, 0.1416]	0.9999

**TABLE A6:** rmANOVA results for Flow Short Scale.

Variable	SS	df [1, 2]	MS	*F*	*p*	*p* (GG)	$\eta_{p}^{2}$	*ε*
Difficulty level	103.458	[2, 24]	51.729	66.775	< 0.001	< 0.001	0.848	0.661
Ergonomic position	1.007	[1, 12]	1.007	3.236	0.097	0.097	0.212	1
Difficulty level * ergonomic position	0.245	[2, 24]	0.123	0.468	0.632	0.576	0.038	0.735

Abbreviation: GG, Greenhouse–Geisser.

**TABLE A7:** Tukey's results HSD for Flow Short Scale.

Condition 1	Condition 2	Mean difference	CI	*p*
Flow	Overload	−2.589	[−2.9192, −2.2581]	< 0.001
Flow	Underload	−2.265	[−2.5958, −1.9348]	< 0.001
Overload	Underload	0.323	[−0.0072, 0.6539]	0.0567

**TABLE A8:** rmANOVA results for NASA‐TLX*.*

Variable	SS	df [1, 2]	MS	*F*	*p*	*p* (GG)	$\eta_{p}^{2}$	*ε*
Difficulty level	22276.374	[2, 24]	11138.187	52.781	< 0.001	< 0.001	0.815	0.857
Ergonomic position	158.278	[1, 12]	158.278	2.252	0.159	0.159	0.158	1
Difficulty level * ergonomic position	317.72	[2, 24]	158.86	2.094	0.145	0.148	0.149	0.949

Abbreviation: GG, Greenhouse–Geisser.

**TABLE A9:** Tukey's results HSD for NASA‐TLX*.*

Condition 1	Condition 2	Mean difference	CI	*p*
Flow	Overload	23.58	[16.9338, 30.2329]	< 0.001
Flow	Underload	−17.67	[−24.3205, −11.0214]	< 0.001
Overload	Underload	−41.25	[−47.9038, −34.6047]	< 0.001

**TABLE A10:** Behavioral and subjective results of pilot study*.*

Measure	Underload [mean ± SD]	Flow [mean ± SD]	Overload [mean ± SD]
Relative score	940.33 ± 348.42*	1884.33 ± 334.20*	305.67 ± 500.52*
NASA‐TLX	19.76 ± 12.36	28.53 ± 14.31	50.63 ± 21.07*
Flow Short Scale	4.38 ± 0.87	5.10 ± 0.91	4.26 ± 0.96

*Note:* All participants completed the experimental procedure as described in the sitting position. In addition to the experimental procedure, participants were interviewed about the primary task design. Based on the results of the NASA‐TLX, the difficulty of the underload condition was decreased to establish a significant difference to the flow condition. Based on the results of the Flow Short Scale and interviews, the overload condition was redesigned to prohibit certain strategies in the overload condition (i.e., the spawning behavior of the red diamonds was adjusted).

*Significantly different from both other conditions at *α* = 0.05.
